# The phosphoinositide 3-kinase inhibitor ZSTK474 increases the susceptibility of osteosarcoma cells to oncolytic vesicular stomatitis virus VSVΔ51 via aggravating endoplasmic reticulum stress

**DOI:** 10.1080/21655979.2021.1999372

**Published:** 2021-12-07

**Authors:** Jinqiong Jiang, Weida Wang, Weineng Xiang, Lin Jiang, Qian Zhou

**Affiliations:** a Department of Oncology, Hunan Provincial People’s Hospital, the First Affiliated Hospital of Hunan Normal University, Changsha, China; b Department of Spine Surgery, The First Hospital of Changsha, Changsha, Hunan, China

**Keywords:** Osteosarcoma, PI3K inhibitor, Oncolytic virus, VSVΔ51, ZSTK474

## Abstract

Blockage of phosphoinositide 3-kinase (PI3K)/protein kinase B (Akt) signal pathway is effective to increase the cytotoxic effects of oncolytic virus on cancer cells, but the detailed mechanisms are still largely unknown. Based on this, the present study managed to investigate the anti-tumor effects of PI3K inhibitor ZSTK474 and oncolytic vesicular stomatitis virus VSVΔ51 combination treatments on osteosarcoma (OS) *in vitro* and *in vivo*. Specifically, ZSTK474 aggravated the inhibiting effects of VSVΔ51 on osteosarcoma development by triggering endoplasmic reticulum (ER)-stress mediated apoptotic cell death. Mechanistically, either ZSTK474 or VSVΔ51 alone had limited effects on cell viability in osteosarcoma cells, while ZSTK474 and VSVΔ51 combination treatments significantly induced osteosarcoma cell apoptosis. Interestingly, VSVΔ51 increased the expression levels of IRE1α and p-PERK to initiate ER stress in osteosarcoma cells, which were aggravated by co-treating cells with ZSTK474. Next, the promoting effects of ZSTK474-VSVΔ51 combined treatment on osteosarcoma cell death were abrogated by the ER-stress inhibitor 4-phenyl butyric acid (4-PBA), indicating that ZSTK474 enhanced the cytotoxic effects of VSVΔ51 on osteosarcoma cells in an ER-stress dependent manner. Finally, the xenograft tumor-bearing mice models were established, and the results showed that ZSTK474-VSVΔ51 combined treatment synergistically hindered tumorigenesis of osteosarcoma cells *in vivo*. Taken together, our data suggested that ZSTK474 was a novel agent to enhance the cytotoxic effects of VSVΔ51 on osteosarcoma by aggravating ER-stress, and the present study might provide alternative therapy treatments for osteosarcoma in clinic.

## Background

Osteosarcoma (OS) is the most common form of bone cancer in children and adolescents, which belongs to a large family of mesenchymal-derived solid tumors with considerable histological, genetic and molecular heterogeneity [1–3]. It is initiated by somatic TP53 and/or RB1 mutations that lead to chromosomal instability and malignant transformation [4]. The 5-year survival rate of patients diagnosed with metastatic or recurrent osteosarcoma is less than 30% [5], which brought huge health burden for human beings. Chemotherapy had widely been used for OS treatment, however, a recent multi-center clinical trial showed that increasing the dose of chemotherapy improved prognosis of patients but did not prolong survival in high-risk populations, which underscored the need for novel treatment strategies [6]. Therefore, development of effective treatment strategies for OS became urgent and necessary.

Oncolytic viruses are a novel therapeutic tool that have shown encouraging outcomes in various cancer models [7], and recent data evidenced that oncolytic virus treatments were also proved to be effective for OS [8,9]. Specifically, the oncolytic herpes virus Talimogene Laherparepvec (T-VEC) was recently approved by FDA for treating metastatic melanoma [10–13]. As one of the members of Rhabdoviridae family, Vesicular stomatitis virus (VSV) exerts its oncolytic activities in preclinical models and is currently used for the treatment of osteosarcoma [14,15]. Site-directed mutagenesis of VSV allows it to preferentially target tumors without infecting the healthy cells [16]. For example, VSVΔ51 can only replicate in tumor cells and not in normal cells due to the methionine deletion at position 51 [17]. Mechanistically, oncolytic viruses infected cancer cells through a dual mechanism of selective replication and lysis, and induced an anti-tumor immune response in the host [18,19]. Although the significant advances had been reached in oncolytic virus therapy, its therapeutic efficacy was still seriously limited [20]. To improve the therapeutic effects of oncolytic virus against solid tumors, combination therapy or genetically engineered viruses are currently being explored [21,22].

According to the previous publications [23–25], oncolytic virus induced cell death in multiple cancers through triggering endoplasmic reticulum (ER) stress, and induction of ER stress mediated cell death by its ‘inducers’ had also been evidenced as an effective therapy treatment in various cancer types [26–28]. The above information rendered the possibility that combination of oncolytic virus and ER stress ‘inducers’ might be a novel strategy to treat OS. Based on the existed information, activation of PI3K/Akt signaling pathway facilitated the development of cancers [29,30], and blockage of this pathway aggravated ER stress [31,32]. Interestingly, PI3K inhibitors and oncolytic virus combination treatments had been proved to be as a reasonable strategy for prostate cancer [33], myeloma [34], glioblastoma [35], etc. Nevertheless, the therapeutic efficacy of this treatment strategy had not been evaluated in OS.

As one of the oncolytic viruses, VSVΔ51 has been used for the treatment of various cancers [36–38], but its therapeutic efficacy is seriously limited as the results of the anti-drug effects of cancer cells. Thus, in this study, we managed to determine whether the PI3K inhibitor ZSTK474 enhanced the oncolytic activity of VSVΔ51 in OS cells, and found that the former acted as a potent adjuvant and significantly increased viral cytotoxicity *in vitro* and *in vivo* by aggravating VSVΔ51-induced ER stress. This study firstly aimed to investigate the potential utilization of ZSTK474-VSVΔ51 combined therapy for OS, and our findings provided alternative therapies for OS in clinic.

## Materials and methods

### Cell lines and reagents

The osteosarcoma cell lines (143B, G-63, Saos-2, U2OS, SJSA-1 and HOS), and the Vero cells were obtained from the American Type Culture Collection (ATCC). RPMI-1640 medium and Dulbecco’s modified Eagle’s medium (DMEM) were purchased from Hyclone (Logan, USA). Fetal bovine serum and L-glutamine were purchased form Gibco (Gaithersburg, USA). Penicillin and streptomycin were obtained from Sangong Biotech (Shanghai, China). HY-50847 were obtained from MCE (NJ, USA). Antibodies against IRE1α, p-ERK and ATF6 were purchased from Santa Cruz Biotechnology (TX, USA). Antibodies against CHOP and Ki-67 were purchased form Cell Signaling Technology (MA, USA). Antibody against caspase-12 were purchased form Abcam (OR, USA). Antibody against GAPDH were purchased form Bioworld (MN, USA).

### Cell culture

The MG-63, Vero and Saos-2 cells were cultured in RPMI-1640 medium, and 143B, U2OS, SJSA-1 and HOS cells in Dulbecco’s modified Eagle’s medium, each supplemented with 10% fetal bovine serum, 2 mM L-glutamine, 100 U/mL penicillin and 100 μg/mL streptomycin. Cells were cultured at 37°C in a humidified incubator with 5% CO_2_.

### VSV production, quantification, and infection

GVP expressing VSVΔD51, a recombinant derivative of the Indiana VSV serotype, was provided by Dr. John Bell and Dr. David Stojdl (Ottawa Health Institute) [17]. VSVΔD51 was cultivated in the Vero cells and virus titers were quantified by the standard plaque analysis method as previously described [39].

### Cell viability assay

The osteosarcoma cell lines (143B, G-63, Saos-2, U2OS, SJSA-1 and HOS) were seeded in a 96-well plate at the density of 3000 cells/well. Following treatment with ZSTK474, thiazole blue (3-(4,5-dimethylthiazol-2-yl)-2,5-diphenyltetrazolium) was added to each well as the final concentration of 1 mg/mL, and the cells were incubated for 3 h at 37°C. The medium was removed and the MTT crystals were dissolved in 100 μL DMSO, and the absorbance at 570 nm was measured using Pickers (iMark, Bio-Rad).

### Western blotting

Cells or tissues were lysed with mammalian protein extraction reagent (M-PER; Thermo Science, USA) and separated by SDS-PAGE. The proteins bands were transferred to immunoblot membranes and probed with antibodies against IRE1α (1:1500, #ab237171, Abcam, UK), p-ERK (1:1000, #ab223500, Abcam, UK), ATF6 (1:2000, #ab203119, Abcam, UK), CHOP (1:1000, #AC532, Beyotime, Shanghai, China), caspase-12 (1:1500, #ab8118, Abcam, China) and GAPDH (1:2500, #ab9484, Abcam, UK). The membrane was developed on a ChemiDoc XRS+ system (Bio-Rad) using Immobilon Western chemiluminescence HRP substrate (Millipore), and the protein bands were quantified using Image J software.

### Transmission electron microscopy

The 143B cells were infected with VSVΔ51 at 0.0011 PFU/cell for 246 h in the presence or absence of ZSTK474. The infected cells were centrifuged at 1000 g for 5 min at room temperature. After washing once with PBS, the cells were pelleted at 1500 g for 5 min, and fixed with 2.5% glutaraldehyde and 2% paraformaldehyde in 0.1 M phosphate buffer (pH 7.4) on ice. The samples were then analyzed by standard transmission electron microscopy.

### Xenograft tumor-bearing mice models

Female BALB/c-nu/nu mice (N = 24, 4 weeks old) were purchased from Research Animal Center of Sichuan Medical College, and were each subcutaneously injected with 5 × 10 ^6^ 143B cells on their dorsal side. Palpable tumors (~50 mm3) appeared within 3 days, and once they reached ~150 mm^3^ after 6 days, the tumor-bearing mice were intravenously injected with VSVΔ51 (2.5 × 107 pfu/kg/d) between 6–8 days, and the mice with VSVΔ51 pre-injection were then treated with or without ZSTK474 (2 mg/kg/d) for a total of 6 times from day 12 to 14. The placebo group was injected with equal volume of PBS at the same time points. The mice were equally divided into 4 groups, including PBS group, ZSTK474 alone group, VSVΔ51 alone group and ZSTK474 + VSVΔ51 group. The length and width of the tumor were measured every 3 d, and the volume was calculated as (length × width^2^)/2. The animal experiments were approved by the Animal Ethics and Welfare Committee of The First Hospital of Changsha (Approval number: 20190507–02).

### Immunohistochemistry

Tumor sections (4 μm thick) were dewaxed in xylene, hydrated in an ethanol gradient, soaked in 0.3% H_2_O_2_-methanol for 30 min, washed with PBS, and incubated overnight with monoclonal antibodies against Ki-67 (9449s, Cell Signaling Technology), IRE1α, CHOP and Cleaved-caspase-12 at 4°C. After washing once, the sections were incubated with biotin-labeled goat anti-rabbit or anti-mouse IgG for 2 h at room temperature. Immunostaining was developed with streptavidin/peroxidase complex and diaminobenzidine, and counterstained with hematoxylin.

### Apoptosis detection

The 143B cells were incubated with PBS, VSVΔ51 (0.01 PFU/cell), VSVΔ51+ ZSTK474 (1 µM) and VSVΔ51+ ZSTK474 + 4-PBA (0.2 mM) for 6 h under the standard culture conditions, and the Annexin V-FITC/PI double staining assay kit (BD Bioscience, Sanjose, CA, USA) was purchased to examine cell apoptosis ratio based on the experimental protocols provided by the manufacturer. The data was collected and analyzed by using the FACSCalibur Flow Cytometer (BD Bioscience, Sanjose, CA, USA).

### Statistical analysis

All statistical analyses were performed using GraphPad Prism software. Different groups were compared with Student’s t-test or analysis of variance (ANOVA) as appropriate. Tumor volumes were calculated by repeated measurement of single factor analysis of variance. *P* values less than 0.05 were considered statistically significant.

## Results

### ZSTK474 sensitizes osteosarcoma cells to VSVΔ51

**Figure 1.**
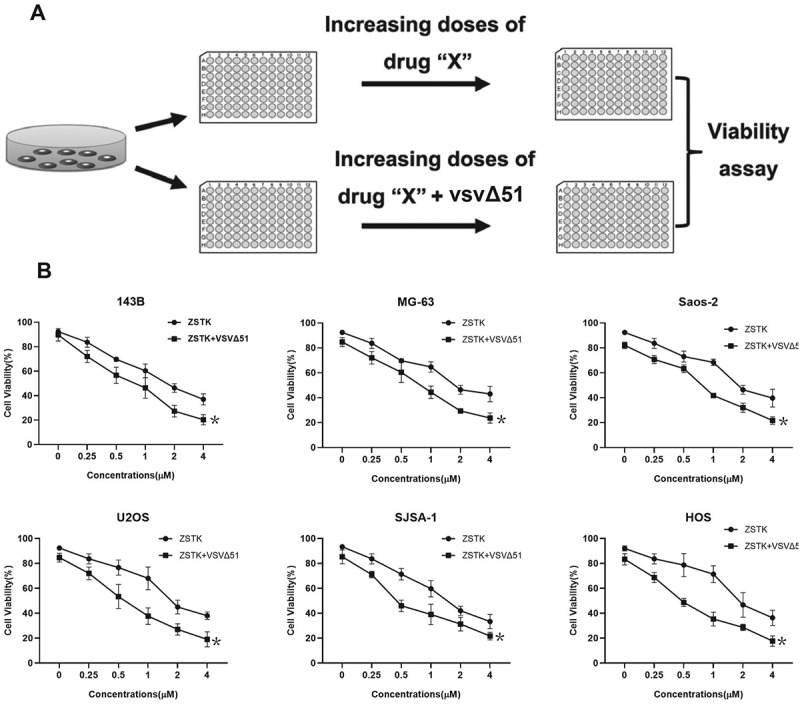
ZSTK474 sensitizes osteosarcoma cells to VSVΔ51. a. experimental outline: 143B, G-63, Saos-2, U2OS, SJSA-1 and HOS cells were treated with varying doses of ZSTK474 with/out VSVΔ51 (MOI = 0.0011). b. percentage of viable cells following different treatments. each experiment repeated at least 3 times

We initially investigated the effects of ZSTK474 and VSVΔ51 on cell viability in the osteosarcoma cells. The OS cells (143B, MG-63, Saos-2, U2OS, SJSA-1 and HOS) were treated with varying doses of PI3K inhibitor (ZSTK474) in the presence/absence of low titer (0.001 PFU/cell) VSVΔ51 oncolytic virus (Figure 1(a)). The MTT assay was performed to determine cell viability, and the results showed that ZSTK474 decreased the viability of OS cells in a dose-dependent manner, and also augmented the cytotoxic effects of VSVΔ51 (*P* < 0.05, Figure 1(b)). Specifically, almost 80% of the infected cells were dead when additionally treated with 4 µM ZSTK474, indicating that ZSTK474 sensitized osteosarcoma cells to VSVΔ51 (Figure 1(b)). Consistently, the data in Figure 3(c) also suggested that ZSTK474-VSVΔ51 combined treatment significantly increased cell apoptosis in OS cells, compared to the VSVΔ51 alone group (*P* < 0.05). Those data suggested that ZSTK474 was capable of enhancing the oncolytic effects of VSVΔ51 virus in osteosarcoma.

**Figure 2.**
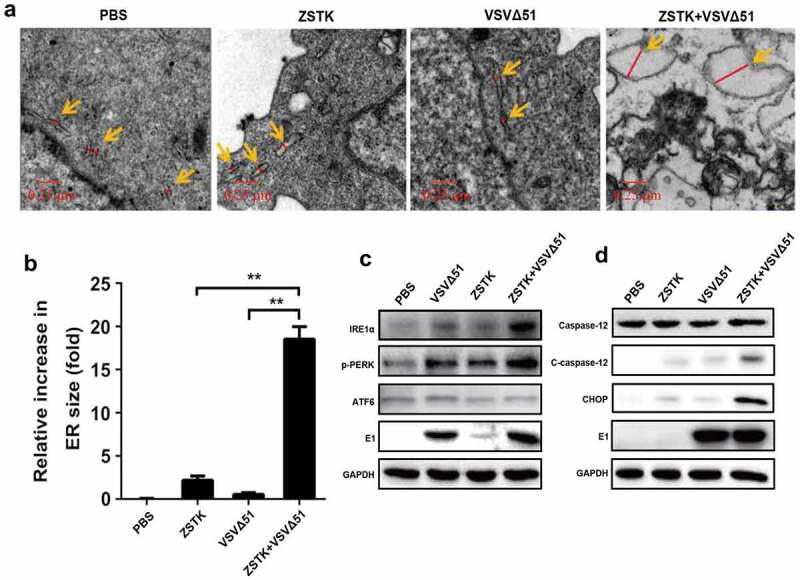
PI3K inhibition aggravated ER stress in VSVΔ51-infected cells. a. transmission electron microscopy (TEM) images of PBS, ZSTK474, VSVΔ51 and ZSTK474 + VSVΔ51-treated 143B cells *in vitro*. Orange arrows indicate the ER and the red line trace the relative size. scale bar = 0.25 μm. b. ER swelling as quantified from TEM images. data is expressed as mean ± SD (n = 4, ** P < 0.01). C-D. Immunoblot showing expression levels of (c) IRE1α, p-PERK and ATF6, and (d) CHOP, caspase-12 and E1 proteins in the differentially treated 143B cells *in vitro*. EACH experiment repeated at least 3 times. **P < 0.01

**Figure 3.**
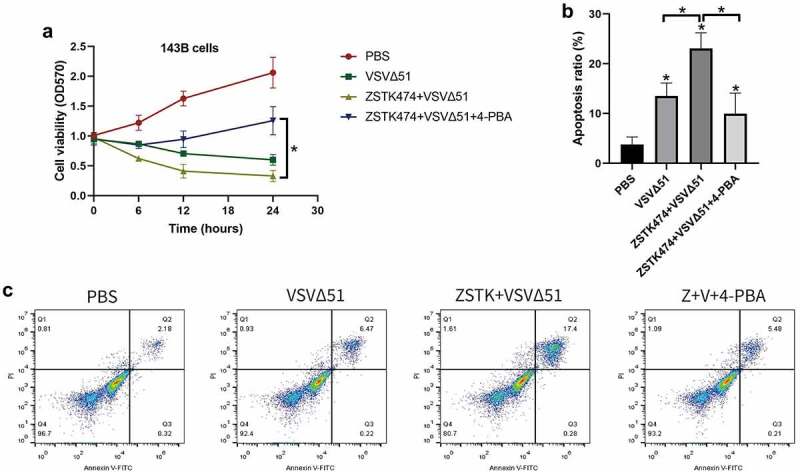
ZSTK474-VSVΔ51 combination treatment triggered ER stress to induce apoptotic cell death in OS cells (143B). a. the OS cells were subjected to PBS, VSVΔ51, VSVΔ51+ ZSTK474 and VSVΔ51 + ZSTK474 + 4-PBA for 0 h, 6 h, 12 h and 24 h, respectively, MTT assay was performed to examine cell viability. b-c. At 6 h post-treatment, the annexin V-FITC/PI double staining assay was conducted to examine cell apoptosis, and the data was visualized by histogram. each experiment repeated at least 3 times. *P < 0.05

### ZSTK474 aggravated VSVΔ51-induced ER stress in osteosarcoma cells

The surge in protein synthesis during viral replication frequently overloads the endoplasmic reticulum, resulting in ER stress that triggers the unfolded protein response (UPR) to restore protein homeostasis and decrease aggregates of misfolded peptides. However, persistent UPR due to severe ERS can lead to apoptosis [40]. In addition, previous data suggested that both oncolytic virus and PI3K inhibitor affected ER stress [26–28]. Thus, we next explored the role of ZSTK474-VSVΔ51 co-treatments in regulating ER stress in osteosarcoma cells. Mechanistically, as shown in Figure 2(a,b), the 143B cells treated with ZSTK474 or VSVΔ51 showed slightly swollen endoplasmic reticulum within 24 h, which were markedly increased by the ZSTK474-VSVΔ51 combination treatment (*P* < 0.05). In addition, the Western Blot analysis results showed that ZSTK474-VSVΔ51 combined treatment synergistically increased the expression levels IRE1α, p-PERK and E1 to induce ER-stress in OS cells (*P* < 0.05, Figure 2(c), Figure S1A). Furthermore, we explored the possible mechanisms, and found that the caspase-12 pathway was strongly activated in the combination treatment group, along with upregulation of C/EBP-homologous protein (CHOP) compared to the ZSTK474 or VSVΔ51-treated cells (*P* < 0.05, Figure 2(d), Figure S1B). However, future work is still needed to investigate this issue. In general, our data evidenced that ZSTK474 augmented VSVΔ51-induced ER stress in OS cells.

### ZSTK474 and VSVΔ51 combination treatments induced cell death in osteosarcoma cells through activating ER stress

Given that ER-stress triggered apoptotic cell death [26–28], and ZSTK474-VSVΔ51 combination treatment triggered ER-stress in OS cells, we next validated that ZSTK474 and VSVΔ51 synergistically inhibited OS progression *in vitro* in an ER-stress dependent manner (Figure 3(a-c)). To achieve this, the 143B cells were pre-treated with ER stress inhibitor 4-PBA, and were divided into four groups, including PBS, VSVΔ51, ZSTK474+ VSVΔ51, and ZSTK474+ VSVΔ51 + 4-PBA groups, respectively. As expected, the MTT assay results showed that ZSTK474 aggravated the inhibiting effects of VSVΔ51 on cell viability in OS cells, and the suppressing effects of ZSTK474-VSVΔ51 co-treatments on cell viability in the osteosarcoma cells were abrogated by co-treating cells with 4-PBA. (*P* < 0.05, Figure 3(a)). Consistently, by performing the Annexin V-FITC/PI double staining assay, we noticed that the promoting effects of ZSTK474-VSVΔ51 combined treatment on cell apoptosis in OS cells were also abrogated by 4-PBA (*P* < 0.05, Figure 3(b,c)), implying that ZSTK474 and VSVΔ51 triggered ER-stress to promote OS cell death.

### ZSTK474 and VSVΔ51 synergistically inhibit tumor growth in vivo

**Figure 4.**
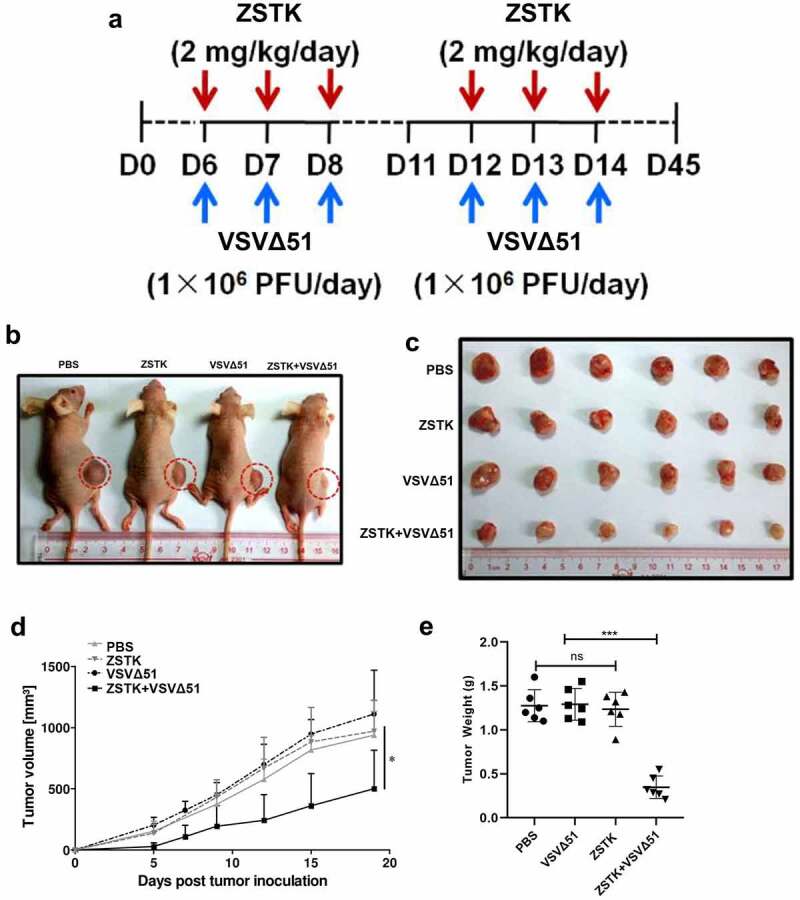
ZSTK474 improved the therapeutic effect of VSVΔ51 in the mouse model of osteosarcoma. a. experimental flowchart. b-c. representative images of tumors from the PBS, ZST K474, VSVΔ51 and combination treatment groups. d. changes in tumor volume in the different groups (n = 5). e. tumor weight in each treatment group at the end of the experiment (n = 5, *** *P* < 0.001)

**Figure 5.**
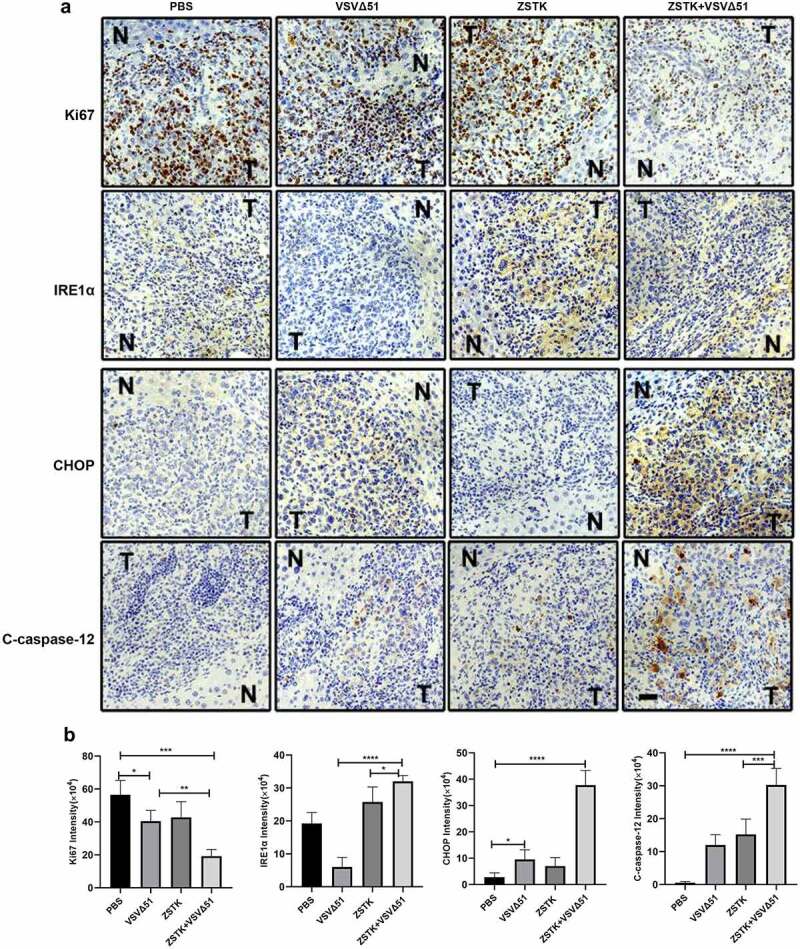
ZSTK474 and VSVΔ51 inhibited osteosarcoma growth xenograft growth *in vivo*. a. in situ expression of Ki-67 (proliferation marker), IRE1α, CHOP and C-caspase-12 in the tumor tissues. scale bar = 50 μm. b. quantification of immunohistochemistry results. Each experiment contained at least 3 repetitions. *** *P* < 0.001

The *in vivo* therapeutic potential of ZSTK474 and VSVΔ51 was finally evaluated in an OS xenograft model, as it outlined in Figure 4(a). Compared to ZSTK474 or VSVΔ51 alone, the combination treatment significantly inhibited tumor growth (*P* < 0.001, Figure 4(b-d)). Furthermore, the tumor weight in the combination treatment group was significantly lower than that in ZSTK474 or VSVΔ51 alone groups (*P* < 0.05, Figure 4(e)). Consistent with this, in situ Ki67 expression was significantly lower, and that of IRE1α, CHOP and C-caspase-12 were higher in the mice tumor tissues of the combination treatment group compared to the ZSTK474 or VSVΔ51 alone-treated tumors (*P* < 0.05, Figure 5(a,b)). Of note, either ZSTK474 or VSVΔ51 alone had no significant effects on the expression levels of Ki67, CHOP and C-caspase-12 (*P* > 0.05, Figure 5(a,b)). However, we noticed that the expression levels of IRE1α were downregulated by VSVΔ51, but were upregulated by ZSTK474 (*P* < 0.05, Figure 5(a,b)). Thus, we validated that inhibition of PI3K by ZSTK474 enhanced viral infection-induced ER stress and synergistically inhibited tumor growth *in vivo*.

## Discussion

Development of novel oncolytic viruses has been proved as a promising immunotherapeutic strategy against osteosarcoma (OS) [8,9] and several of them had been used for the treatment of this type of cancer [14,15]. Currently, FDA approves T-Vec developed by Angen in October 2015 for the treatment of metastatic melanoma [10]. The main focus of oncolytic virus therapy is to directly kill the tumor cells as well as activate the anti-tumor immune response [11]. Genetically engineered recombinant oncolytic viruses have also been designed to improve antitumor activity [41]. However, development of this strategy is limited by the small viral genomes and technical difficulty. In addition, excessive modification of the virus skeleton reduces its infection ability, and therefore oncolytic function [42]. Thus, recent studies are mainly focusing on ‘conditional enhancement’ the efficacy of oncolytic viruses through synergistically using pharmacological agents [43].

The PI3K-AKT-mTOR signaling pathway is frequently dysregulated during tumor initiation and progression [29,30]. Studies show that PI3K inhibitors increase tumor cell apoptosis and modulate the tumor immune microenvironment and immune-infiltrating cells [44,45]. In addition, recent data suggest that blockage of the PI3K-AKT-mTOR pathway by its inhibitors aggravate ER stress in various cells [31,32], and Lei Yang et al. validate that the PI3K/Akt pathway involves in regulating ER stress in granulosa cells [46]. In addition, there exist an interplay between ER stress and autophagy [47,48], and researchers noticed that palmitic acid induced cell death through modulating ER stress and autophagy [47]. Interestingly, induction of ER stress is evidenced as an effective strategy to improve the oncolytic activities of oncolytic virus [26–28]. Furthermore, given that the PI3K inhibitor (ZSTK474) exerted its anti-tumor effects in human sarcoma cells [49], the present study focused on investigating the therapeutic effects of ZSTK474 combined with the oncolytic virus (VSVΔ51) in OS. As expected, by conducting *in vitro* experiments, we found that ZSTK474 aggravated the promoting effects of VSVΔ51-induced ER-stress, resulting in apoptotic cell death in OS cells. Consistently, the *in vivo* data verified that ZSTK474- VSVΔ51 combination treatment had better anti-tumor activities in xenograft mice models, in contrast with the VSVΔ51 alone group.

Our findings provided a strong experimental basis for using small molecule inhibitors of AKT and mTOR as adjuvants along with oncolytic virus to enhance therapeutic effects against solid tumors. However, there are still some limitations in our study that ought to be addressed in our future work. For example, we injected the oncolytic virus via the intravenous route to ensure tumor-specific infection of the oncolytic viruses, but previous studies show that intra-tumoral or peritumoral injection result in better anti-tumor effects [50]. Therefore, the optimum dosage regimen needs to be further explored in the follow-up experiments.

## Conclusions

Taken together, by conducting preliminary experiments, the present study firstly investigated the possibility of PI3K inhibitor (ZSTK474) and oncolytic virus (VSVΔ51) combination treatments for OS therapy, and found that ZSTK474 enhanced the cytolytic effects of VSVΔ51 on OS cells *in vitro* and *in vivo* in an ER-stress dependent manner. These findings evidenced that ZSTK474-VSVΔ51 might be a novel strategy for OS treatment in clinic.

## Supplementary Material

Supplemental Material
