# Supplementary material for: The phosphoinositide 3-kinase inhibitor ZSTK474 increases the susceptibility of osteosarcoma cells to oncolytic vesicular stomatitis virus VSVΔ51 via aggravating endoplasmic reticulum stress
Source: Bioengineered. 2021 Dec 7;12(2):11847–57. doi: 10.1080/21655979.2021.1999372 (PMC8809975; doi:10.1080/21655979.2021.1999372)
Supplement: Supplemental Material [file KBIE_A_1999372_SM4524.docx]

**Supplementary Figures and Figure legends**


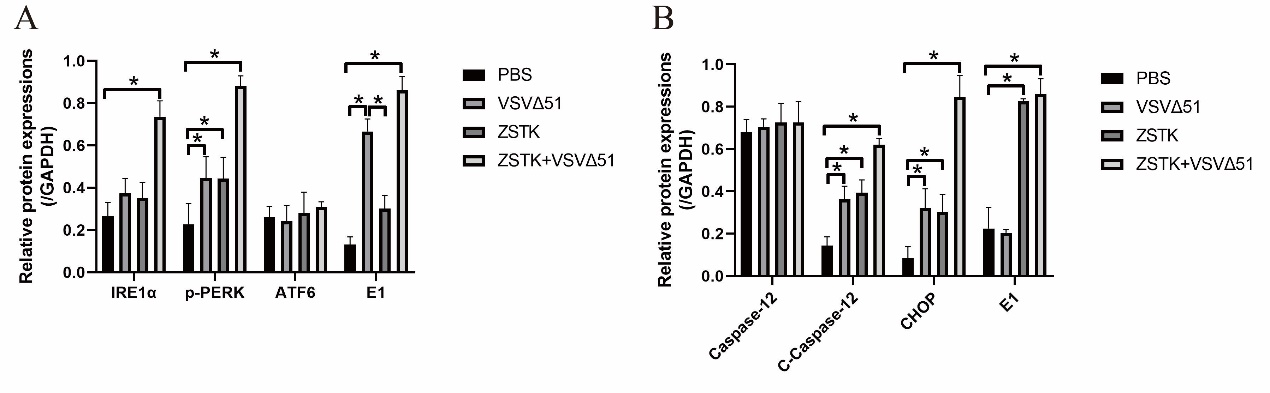


Figure S1. Quantification of protein expressions by grey values in (A) Figure 2C and (B) Figure 2D. The target proteins were normalized to GAPDH. **P* < 0.05.
